# Supplementary material for: Spectral Probe for Electron Transfer and Addition Reactions of Azide Radicals with Substituted Quinoxalin-2-Ones in Aqueous Solutions
Source: Int J Mol Sci. 2021 Jan 10;22(2):633. doi: 10.3390/ijms22020633 (PMC7828026; doi:10.3390/ijms22020633)
Supplement: Supplementary file 1 [file ijms-22-00633-s001.pdf]

Supporting Information for

Spectral Probe for Electron Transfer and Addition Reactions of Azide Radicals with Substituted Quinoxalin-2-ones in Aqueous Solutions

Konrad Skotnicki [1], Slawomir Ostrowski [2], Jan Cz. Dobrowolski [2], Julio R. De la Fuente [3], Alvaro Cañete [4] and Krzysztof Bobrowski [1]**

[1] Centre of Radiation Research and Technology, Institute of Nuclear Chemistry and Technology, 03-195 Warsaw, Poland

[2] Centre of Radiochemistry and Nuclear Chemistry, Institute of Nuclear Chemistry and Technology, 03-195 Warsaw, Poland

[3] Departamento de Química Organica y Fisicoquímica, Facultad de Ciencias Químicas y Farmaceuticas, Universidad de Chile, Casilla 223, Santiago 1 8380492, Chile

[4] Instituto de Ciencias Químicas Aplicadas, Universidad Autónoma de Chile, Santiago, Chile

Contents:

Figure S1. Ground-state absorption spectra of 7-R-3-MeQ derivatives in aqueous solutions containing 0.1 mM 7-R-3-MeQ at pH 7 (A) and pH 11.3 (B)

S3

Figure S2. Corrected for the ground-state absorption of 3-MeQ transient absorption spectra recorded in aqueous solutions containing 0.1 mM 3-MeQ at pH 7

S3

Figure S3. Transient absorption spectra recorded 10 μs after the electron pulse in N_2O-saturated aqueous solutions containing 0.1 mM 7-NH_2-3-MeQ and 0.1M NaN_3 at pH 7

S4

Figure S4. Acid-base equilibria of 7-R-3-MeQ derivatives in the investigated pH range

S4

Figure S5. Time profiles representing growth of transient absorptions at λ = 530 nm recorded after the electron pulse in N_2O-saturated aqueous solutions at pH = 7 containing 0.1 M NaN_3 and various concentrations of 7-OCH_3-3-MeQ

S4

Figure S6. Time profiles representing growth of transient absorptions at λ = 530 nm recorded after the electron pulse in N_2O-saturated aqueous solutions at pH = 11.3 containing 0.1 M NaN_3 and various concentrations of 7-OCH_3-3-MeQ

S5

Figure S7. Comparison of the transient absorption spectrum recorded in Ar-saturated aqueous solutions containing 0.1 mM 7-OCH_3-3-MeQ, 0.1 M $K_2S_2O_8$ and 0.5 M tert-BuOH at pH = 4 and 7 and the u-ωB97XD/ aug-cc-pVTZ calculated UV-Vis spectra of the 7-OCH_3-3-MeQ$^{\bullet+}$ and 7-OCH_3-3-MeQ$^{\bullet}$ species.

S5

Figure S8. The structures of the $N_3^{\bullet}$ adducts at the C2 carbon atoms (a) and the C3 carbon atoms (b) in 3-MeQ

S5

Table S1. Reduction potentials of inorganic redox couples used in the study

S6

Table S2. pK_a values of the acid-base equilibria of 7-R-3-MeQ derivatives

S6

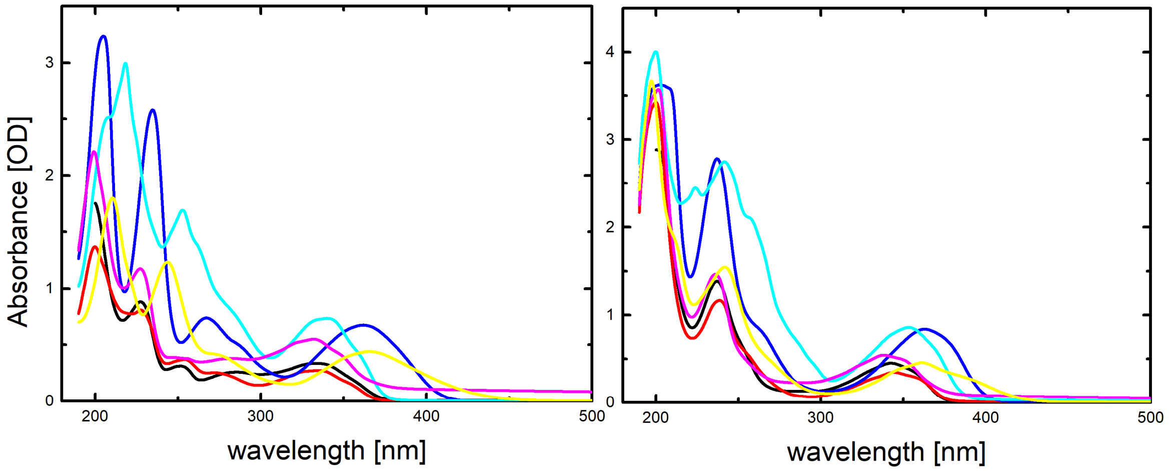

Figure S1. Ground-state absorption spectra of 7-R-3-MeQ derivatives: −H (▬), −CF_3 (▬), −OCH_3 (▬), −CN (▬), −F (▬), −NH_2 (▬) in aqueous solutions containing 0.1 mM 7-R-3-MeQ at pH 7 (A) and pH 11.3 (B)

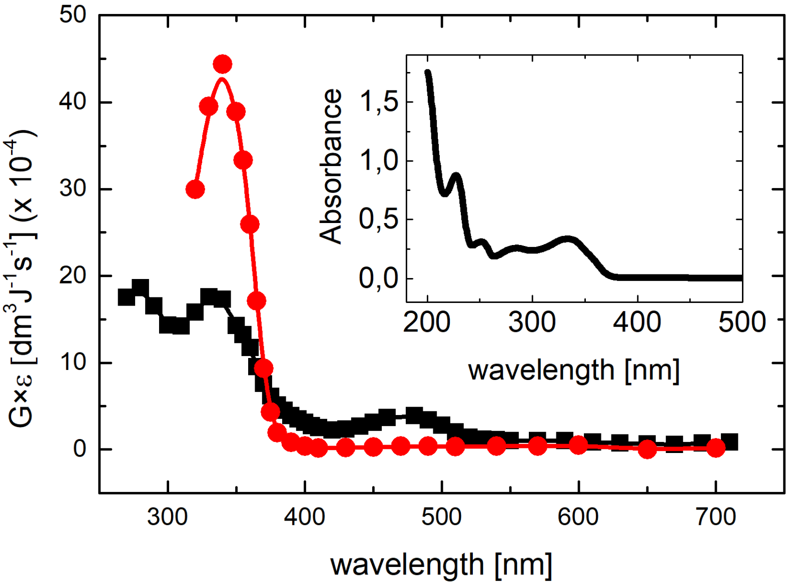

Figure S2. Corrected for the ground-state absorption of 3-MeQ transient absorption spectra recorded in aqueous solutions containing 0.1 mM 3-MeQ at pH 7: (■) 10 μs after the electron pulse in Ar-saturated containing 0.1 M $K_2S_2O_8$ and 0.5 M *tert*-BuOH and (■) 3 μs after the electron pulse in N_2O-saturated and containing 0.1M NaN_3. Inset: Ground-state absorption spectrum of 3-MeQ in aqueous solutions containing 0.1 mM 3-MeQ, at pH 7.

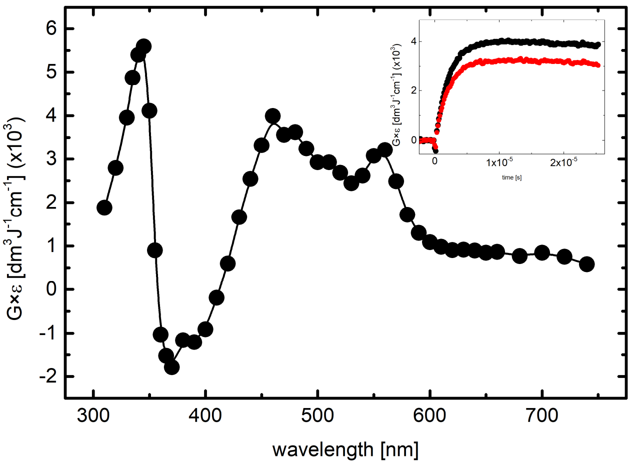

Figure S3. Transient absorption spectra recorded 10 μs after the electron pulse in N_2O-saturated aqueous solutions containing 0.1 mM 7-NH_2-3-MeQ and 0.1M NaN_3 at pH 7. Inset: Time profiles representing growth of transient absorptions at λ = 460 nm (●) and 530 nm (●).

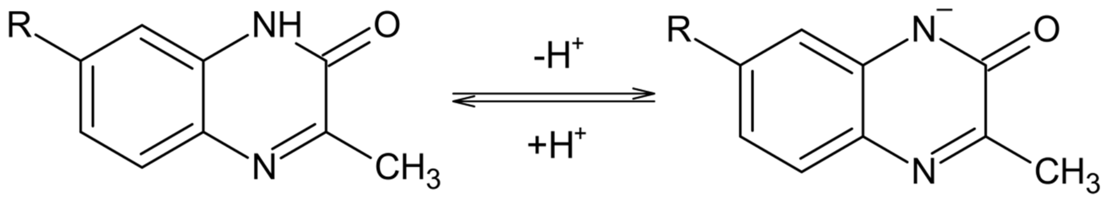

Figure S4. Acid-base equilibria of 7-R-3-MeQ derivatives in the investigated pH range.

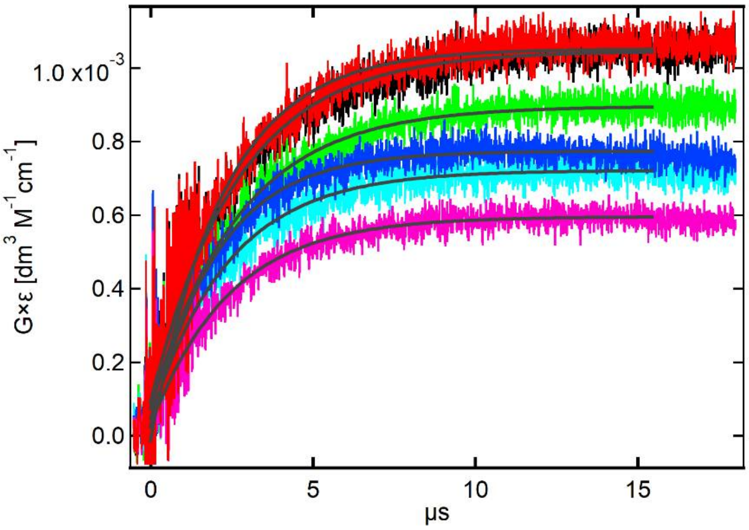

Figure S5. Time profiles representing growth of transient absorptions at λ = 530 nm recorded after the electron pulse in N_2O-saturated aqueous solutions at pH = 7 containing 0.1 M NaN_3 and various concentrations of 7-OCH_3-3-MeQ: (▬) 0.05 mM, (▬) 0.1 mM, (▬) 0.2 mM, (▬) 0.3 mM, (▬) 0.4 mM and (▬) 0.5 mM.

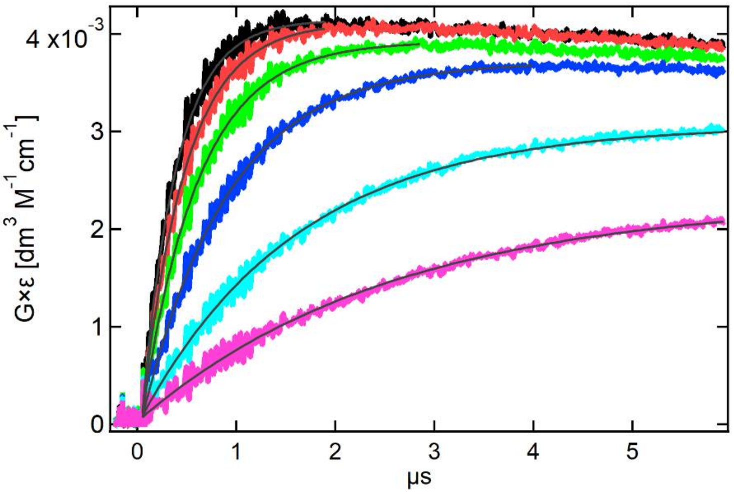

Figure S6. Time profiles representing growth of transient absorptions at λ = 530 nm recorded after the electron pulse in N_2O-saturated aqueous solutions at pH = 11.3 containing 0.1 M NaN_3 and various concentrations of 7-OCH_3-3-MeQ: (▬) 0.05 mM, (▬) 0.1 mM, (▬) 0.2 mM, (▬) 0.3 mM, (▬) 0.4 mM and (▬) 0.5 mM

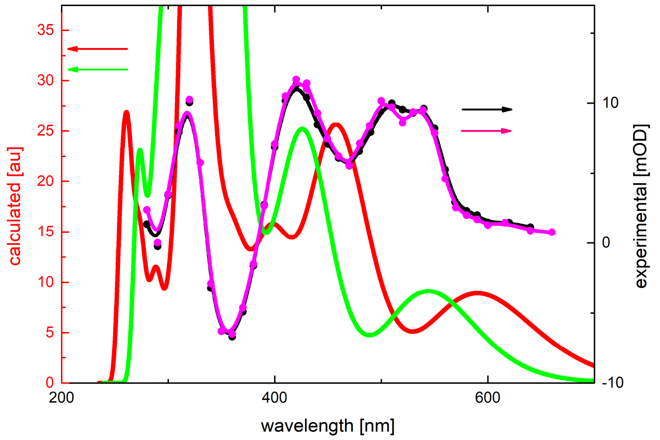

Figure S7. Comparison of the transient absorption spectrum recorded in Ar-saturated aqueous solutions containing 0.1 mM 7-OCH_3-3-MeQ, 0.1 M $K_2S_2O_8$ and 0.5 M tert-BuOH at pH = 4 (●, ▬) and 7 (●, ▬) and the ωB97XD/ aug-cc-pVTZ calculated UV-Vis spectra of the 7-OCH_3-3-MeQ$^{•+}$ (▬) and 7-OCH_3-3-MeQ$^{•}$ (▬) species. Calculated spectra are shifted by 130 nm towards longer wavelengths.

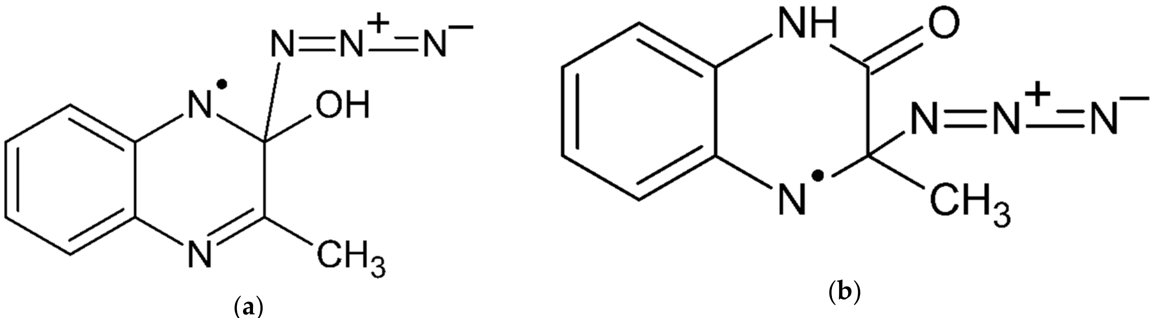

(**a**) (**b**)

Figure S8. Structures of the $N_3^{•}$ adducts at the C2 carbon atoms (**a**) and the C3 carbon atoms (**b**) in 3-MeQ

Table S1. Reduction potentials of inorganic redox couples used in the study

Redox couple	E^0 (V) [1]
$SO_4^{\bullet-}$/ SO_4^{2-}	+2.437 ± 0.019
Tl^{2+}/ Tl^{+}	+2.225 ± 0.007
$CO_3^{\bullet-}$/ CO_3^{2-}	+1.57 ± 0.03
$N_3^{\bullet}$/ N_3^{-}	+1.33 ± 0.01
$(SCN)_2^{\bullet-}$/ 2 SCN^{-}	+1.30 ± 0.02

[1]measured vs. Normal Hydrogen Electrode

Table S2. pK_a values of the acid-base equilibria of 7-R-3-MeQ derivatives

Substituent	pK_a [1]
–CN	8.3
$–CF_3$	9.1
–F	9.1
–H	9.4
$–OCH_3$	9.6
$–NH_2$	10.0

[1] measured in this work
